# Changes in Diversity and Community Composition of Root Endophytic Fungi Associated with *Aristolochia chilensis* along an Aridity Gradient in the Atacama Desert

**DOI:** 10.3390/plants11111511

**Published:** 2022-06-05

**Authors:** María José Guevara-Araya, Víctor M. Escobedo, Valeria Palma-Onetto, Marcia González-Teuber

**Affiliations:** 1 Departamento de Química Ambiental, Facultad de Ciencias, Universidad Católica de la Santísima Concepción, Concepción 4090541, Chile; mguevara@isett.cl (M.J.G.-A.); vpalma@ucsc.cl (V.P.-O.); 2 Centro de Ecología Integrativa, Instituto de Ciencias Biológicas, Universidad de Talca, Talca 3460000, Chile; victor.escobedo@utalca.cl; 3 Instituto de Investigación Interdisciplinaria, Universidad de Talca, Talca 3460000, Chile

**Keywords:** root-associated fungi, endophyte diversity, aridity, soil water availability, Atacama Desert

## Abstract

Despite the widespread occurrence of fungal endophytes (FE) in plants inhabiting arid ecosystems, the environmental and soil factors that modulate changes in FE diversity and community composition along an aridity gradient have been little explored. We studied three locations along the coast of the Atacama Desert in Chile, in which the plant *Aristolochia chilensis* naturally grows, and that differ in their aridity gradient from hyper-arid to semi-arid. We evaluated if root-associated FE diversity (frequency, richness and diversity indexes) and community composition vary as a function of aridity. Additionally, we assessed whether edaphic factors co-varying with aridity (soil water potential, soil moisture, pH and nutrients) may structure FE communities. We expected that FE diversity would gradually increase towards the aridity gradient declines, and that those locations that had the most contrasting environments would show more dissimilar FE communities. We found that richness indexes were inversely related to aridity, although this pattern was only partially observed for FE frequency and diversity. FE community composition was dissimilar among contrasting locations, and soil water availability significantly influenced FE community composition across the gradient. The results indicate that FE diversity and community composition associated with *A. chilensis* relate to differences in the aridity level across the gradient. Overall, our findings reveal the importance of climate-related factors in shaping changes in diversity, structure and distribution of FE in desert ecosystems.

## 1. Introduction

All terrestrial plants in natural ecosystems are considered to harbor fungal endophytes (FE) in their tissues. In most plant species, except for some grasses, FE are horizontally transmitted through spores, having the capacity to colonize a wide range of hosts [1]. Horizontally transmitted FE are abundant and highly diverse in plants [2,3,4,5] and may benefit their hosts by improving growth and resistance to diverse biotic and abiotic factors [1]. FE diversity is strongly influenced by numerous host-related and environmental factors, such as host genotype, host structural and chemical traits, precipitation, temperature and soil chemistry [6,7,8].

FE abundance and diversity usually vary across the latitude and an annual rainfall gradient, decreasing from the tropics to boreal and arctic regions [9,10]. In the tropics and semi-arid regions, FE diversity is higher during the wet seasons in comparison to dry seasons [11,12,13]. Thus, annual precipitation appears to be a crucial factor for determining FE diversity and community composition in different ecosystems [6,14,15,16]. For example, for the tropical tree *Metrosideros polymorpha* (Myrtaceae) in Hawaii, rainfall was one of the key factors structuring foliar FE communities across the landscape [6]. Moreover, Giauque and Hawkes [17] reported that past and present precipitation levels were the most important predictor factors of FE communities in savanna grasslands across a steep precipitation gradient. Few studies have characterized FE diversity and communities in desert environments [18,19]; particularly, how they vary as a function of precipitation. Understanding variations in FE diversity and composition in desert environments is relevant to predicting shifts in the distribution range of FE in contrasting arid environments under climate change.

Deserts are characterized by extreme environmental conditions, where water deficit is one of the major limiting factors constraining species distribution and diversity [20]. Soil microbial diversity (bacteria and fungi), in general, declines towards the aridity gradient increases [21]. Some edaphic factors such as soil water availability, pH and nutrients may change in response to the long-term effects of the climate resulting from aridity gradients [22]. Importantly, they also seem to be relevant in influencing FE diversity in deserts [19]. For example, a study in the Sonora Desert on foliar FE diversity showed that endophytic fungi were isolated approximately 11- to 40-fold less frequently than that expected by the latitude of the study sites [19], suggesting that edaphic factors related to arid environments restrict endophyte distribution. Whereas edaphic factors appear to highly influence variations in soil microbial composition [23], the importance of these factors shaping FE communities at a large scale is not fully understood [24]. In contrast with the numerous studies on foliar FE communities in contrasting environmental and/or geographic sites, studies on the changes in diversity and community composition of root FE along an aridity gradient are still limited [25].

**Figure 1:**
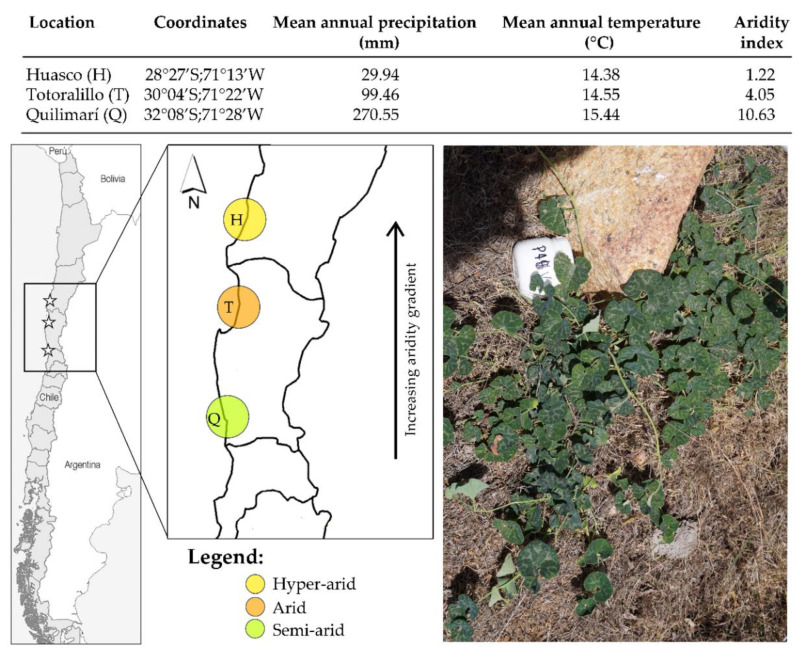
Geographic positions, mean annual precipitation, mean annual temperature and aridity index of selected *Aristolochia chilensis* locations. Locations (H: Huasco; T: Totoralillo; Q: Quilimarí) were chosen based on increasing aridity, ranging from hyper-arid to semi-arid. The annual averages of precipitations and temperature were calculated by taking into account the weather of a 15-year period (data extracted from [26]). The aridity index was calculated according to the De Martonne aridity index [27], where the lowest value represents the most arid place. A picture of a plant of *A. chilensis* growing naturally in the field is shown.

Here, we worked in three locations along the coast of the Atacama Desert in Chile, in which the perennial herb *Aristolochia chilensis* naturally grows, and that differ in their aridity gradient from hyper-arid to semi-arid (Figure 1). Using a culture-dependent method, we characterized the diversity and community composition of FE associated with the roots of *A. chilensis*, growing naturally across the aridity gradient. The aims of the study were to: (1) evaluate how root FE diversity varies along the aridity gradient; (2) determine the degree of structural similarity in root FE communities across locations; and (3) reveal if edaphic factors co-varying with aridity (soil moisture, soil water potential, pH and nutrients) drive the structure of root FE communities. Since FE distribution is restricted by precipitation, it is expected that root FE diversity is lower in the more arid location and that it gradually increases towards the aridity gradient declines. We also predicted that locations that have the most contrasting environments (hyper-arid vs. semi-arid) would show a more different root FE community composition. A manipulative field experiment was additionally performed to assess potential variation in root FE abundance as a function of water availability.

## 2. Results

### 2.1. Diversity and Community Composition of Root Endophytic Fungi

**Figure 2:**
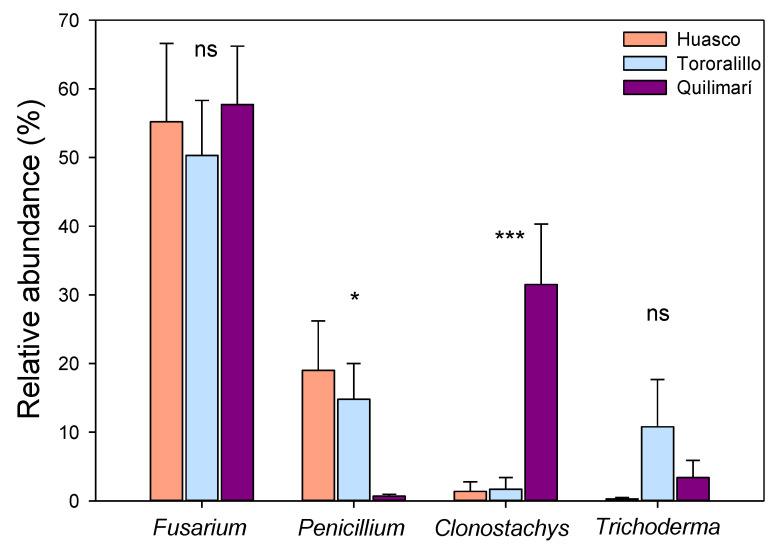
Relative abundance (average ± SE, *n* = 10 plants per location) of the four most abundant root FE genera found in the three locations of *Aristolochia chilensis* (Kruskal–Wallis). * *p* < 0.05, *** *p* < 0.001, ns indicates non-significant differences.

Molecular data revealed that the roots of *A. chilensis* coming from the three locations were colonized by numerous FE taxa. A total of 570 fungal isolates were purified into individual cultures, with a total species richness of 9 OTUs described for the location of Huasco, 29 OTUs for Totoralillo and 10 OTUs for Quilimarí (Table 1). Culture-based methods may underestimate the richness and misrepresent the taxonomic composition of endophyte communities relative to culture-independent approaches, such as PCR product pyrosequencing. Nevertheless, comparative studies suggest that many of the proportionally dominant microbial taxa identified by culture-independent approaches are accurately represented by culture-based techniques [28,29]. In general, the rarefaction curves for locations achieved saturation (Figure S1). For Huasco and Quilimarí, the rarefaction curves tended to be flat, indicating that most FE were covered in every sample of this study. For the location of Totoralillo, however, it was observed that the saturation curve was not completely flattened out, indicating that there were species not covered by the method. Root fungal endophyte communities were mainly dominated by the genera *Fusarium*, *Penicillum*, *Clonostachys,* and *Trichoderma*, which were found to dominate distinct locations in different ways (Figure 1). Both *Fusarium* and *Trichoderma* were similarly abundant among the locations (*Fusarium*: *x*^2^ = 3.20, *p* = 0.201, Kruskal–Wallis; *Trichoderma*: *x*^2^ = 3.36, *p* = 0.186, Kruskal–Wallis). For the *Penicillium* genus, its relative abundance was significantly higher in the more arid locations (Huasco and Totoralillo) than in the wettest location (Quilimarí) (*x*^2^ = 7.60, *p* = 0.022, Kruskal–Wallis), whereas the relative abundance of *Clonostachys* was dominant in the wettest location (Quilimarí) (*x*^2^ = 14.7, *p* = 0.000, Kruskal–Wallis) (Figure 2).

FE frequency (*x*^2^ = 13.31, *p* = 0.001, Kruskal–Wallis), as well as the indexes of Shannon (*x*^2^ = 6.57, *p* = 0.003, Kruskal–Wallis), Chao (*x*^2^ = 23.48, *p* < 0.001, Kruskal–Wallis) and Bootstrap (*x*^2^ = 23.48, *p* < 0.001, Kruskal–Wallis) were significantly different among locations (Table 2). In contrast, no significant differences in the Simpson index were observed among three locations (*x*^2^ = 2.57, *p* = 0.28, Kruskal–Wallis) (Table 2).

**Figure 3:**
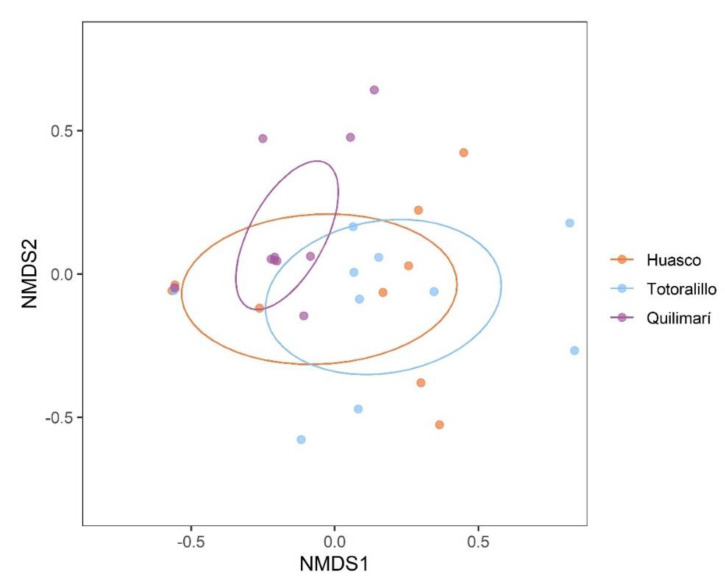
Comparison of fungal endophyte communities in three locations of *Aristolochia chilensis*: Huasco, Totoralillo and Quilimarí. A two-dimensional non-metric multidimensional scaling (NMDS) is shown.

The PERMANOVA indicated that FE community composition significantly differed among locations (Table 3). The pairwise analyses revealed that whereas FE community was similar between Huasco (hyper-arid location) and Totoralillo (arid location), FE community from both Huasco and Totoralillo was significantly different from that from Quilimarí (semi-arid location) (Table 3). This was visualized with the NMDS that showed a grouping in FE communities among locations (Figure 3).

### 2.2. Edaphic Factors and Location Differences

The edaphic factors were significantly different among locations (Table S1). A distance-based redundancy analysis (db-RDA) showed that FE communities were structured by soil water potential and soil moisture (Table 4). The structuring edaphic factors are explained in the first two axes in 65.67% and 34.33% of the model, and its total variance is 13.14, constricted 1.35, without restrictions 11.78.

### 2.3. Field Experiment

FE frequency (total fungal isolates) significantly differed between watered and non-watered plants (x^2^ = 3.86, *p* = 0.043, Kruskal–Wallis). It was observed that the FE frequency of watered plants (36.8 ± 5.9) was two times the frequency of non-watered plants of *A. chilensis* (15.6 ± 2.4).

## 3. Discussion

This study provides evidence that variations in root FE diversity and community composition associated with the endemic species *A. chilensis* relate to differences in the aridity level along the gradient. Our results showed that: (1) Both FE frequency and diversity were considerably lower in the more arid location (Huasco) than in the wettest locations (Totoralillo and Quilimarí); (2) FE richness indexes gradually increase towards the aridity gradient declines; and (3) FE communities were dissimilar across the aridity gradient, and soil water availability was a key factor in structuring FE community composition. Additionally, the abundance distribution of certain dominant FE taxa changed across the gradient, suggesting the importance of some fungal genera in structuring FE communities.

Our proposed pattern, that root FE diversity gradually decreases when the aridity index increases (H < T < Q) was partially reached. The richness indexes fulfilled the expected pattern; Chao and Bootstrap indexes were observed that significantly decreased as the aridity increased. Contrary, FE frequency and the Shannon diversity did not wholly follow this pattern. As expected, both were significantly lower in the hyper-arid location of Huasco; nevertheless, both showed their highest values in the arid location of Totoralillo. Interestingly, Totoralillo showed the highest soil water availability, even when it did not experience the highest level of precipitation and the lowest aridity index. The latter is likely associated with microclimate conditions, highlighting the importance of integrating both local and regional environmental factors in studying FE community composition. Our results concur with previous studies, which showed that marked aridity gradients or distinct aridity conditions modulate rhizosphere and endosphere fungal diversity and community composition [25,30,31]. For example, there is evidence that foliar FE richness in the annual plant *Brachypodium* sp. was greater in wetter sites (over 1000 mm of annual precipitation) than in the drier sites along an aridity gradient (less than 500 mm of annual precipitation) [25]. By contrast, for the cactus *Opuntia ficus-indica*, it was shown that alpha fungal diversity in the rhizosphere and endosphere did not change along a bioclimatic gradient from 1200 to 200 mm of annual precipitation [31]. However, the aridity gradient significantly contributed to differences in fungal species composition (beta diversity) [31].

We found that the locations of *A. chilensis*, which had the most contrasting environments, showed dissimilar root FE communities. Whereas the more arid locations along the gradient (Huasco and Totoralillo) showed similar FE communities, both showed dissimilar FE communities to the wettest location (Quilimarí). Thus, aridity appears to play a key role in driving FE species turnover. Consistently, our data revealed a significant variation in FE community composition as a function of soil water potential and soil moisture. These results agree with previous observations by Herrera, et al. [32], who showed that the abundance of root endophytic fungi associated with the grass *Bouteloua gracilis* considerably increased under irrigation. Soil moisture is likely one of the major factors determining soil microbial communities [33], suggesting that soil moisture pulse events stimulate the activity of soil microbes [34,35]. Moreover, the results of our field experiment (in the Northern location along the gradient) supports these findings, showing that a greater soil water availability was associated with a higher frequency of root FE. Even though pH and nutrients are likely associated with inter-site variation in soil archaeal and bacterial communities [36,37], here we found that neither pH nor nutrients shaped root FE communities.

Our study identified FE taxa that are specific for each location. Whereas *Fusarium* and *Trichoderma* seem to be generalist endophytes, i.e., with the ability to colonize locations with contrasting conditions of aridity, *Penicillium* and *Clonostachys* were observed to be habitat-specific FE taxa. *Penicillium* was dominant in the more arid locations (30 and 100 mm of annual precipitation, respectively), while the opposite pattern occurred for *Clonostachys*, which was almost absent in the more arid locations, but was dominant in the wettest location (over 250 mm of annual precipitation). Thus, our data suggest that extreme aridity selects for specific FE taxa, which likely contribute to variations in FE community composition across the gradient. Some of these taxa such as *Fusarium*, *Penicillium* and *Trichoderma* have previously been reported as common endophytes in plants inhabiting arid and semi-arid ecosystems [18,19,38]. The genus *Penicillium*, for example, has been found to be a dominant FE in below-ground tissues of other plant species native to the Atacama Desert in Chile, including *Chenopodium quinoa* and *Prosopis chilensis* [39,40]. FE isolated from the Atacama Desert seem to be a promising strategy to alleviate abiotic stress in host plants [39,40,41]. Therefore, this study contributes to the identification of new FE strains with potential benefits for plants to survive and tolerate stressful conditions in deserts. Understanding how arid-adapted FE taxa relate to the ability of *A. chilensis* to tolerate extreme arid conditions in the Atacama Desert is key to predicting the ecological significance of root FE in deserts. Further research in this context should consider testing potential differences in host stress tolerance depending on the symbiotic interaction with different aridity-adapted FE strains. Desert environments, despite the hostile conditions (low water availability and poor nutrient conditions), appear to harbor rich rhizosphere and endosphere fungal communities [30,42]. Our findings showed that environmental precipitation and soil water availability are relevant factors contributing to FE diversity and community composition in *A. chilensis*, revealing the importance of climate-related factors in shaping FE structure and distribution in desert ecosystems. Although this research covered only a few plant individuals in each location, our data supported our main prediction, that FE diversity and community composition vary as a function of aridity. Studies on changes in community composition of root FE along an aridity gradient are still limited [25]. Thus, our study provides new evidence in the topic and highlights the importance of considering environmental gradients as an appropriate approach to understand changes in the diversity, and the structure of FE communities. Large-scale studies across climate gradients, to investigate species adaptations to environmental pressures, are becoming common [43,44], and seem to be a promising alternative for climate change research. Understanding how FE community composition varies as a function of precipitation in deserts is critical to comprehending the whole ecosystem function in the face of climate change. The latter is particularly important for the Atacama Desert, the driest temperate desert on Earth [45], where the current decrease in precipitation and increase in temperature are the major threats of species distribution and overall diversity.

## 4. Materials and Methods

### 4.1. Study Species and Study Sites

*Aristolochia chilensis* (Aristolochiaceae) is an endemic perennial plant species in Chile. It is a creeping herb with purple-brownish flowers and dark green leaves [46], and with a pivoting root fragmented into two or more parts, which are several inches long. Although *A. chilensis* has a wide environmental gradient distribution along Chile, its distribution is critically threatened by the effects of human activity, currently showing small and fragmented populations throughout its distribution range (González-Teuber, personal observations). It is found from the North of the Atacama Desert (~27°30′ S) to the Mediterranean regions (~33°29′ S) in central Chile [47]. In this study, roots of *A. chilensis* plants were collected in three natural populations along the Chilean coast, covering parts of the Atacama Desert. The locations selected for this study were Huasco (H), Totoralillo (T), and Quilimarí (Q), which considerably differ in their annual precipitations and aridity index [48,49]; and range from a hyper-arid environment to a semi-arid environment (Figure 1). *A. chilensis* is an appropriate plant system to work along an aridity gradient in Chile, since it is able to colonize hyper-arid sites in the North of Chile, and further spreads to semi-arid and Mediterranean sites more to the South [50]. 

### 4.2. Isolation of FE

Root material was collected in each population of *A. chilensis* (*n* = 10 plants per location). Three pivotal roots of ten healthy plants (without apparent damage from herbivores and pathogens) were collected in November/December 2015 in Huasco, Totoralillo and Quilimarí. The three pivotal roots were pooled in order to increase the number of fungal species isolated. After collection, root material was immediately transported to the lab for further endophyte isolation and molecular characterization. Roots were first washed under running water to remove dirt. Then, the root surface was disinfected in a sequence of washes with ethanol (70%), sodium hypochlorite (1%) and sterilized water (following the protocol described by Arnold, et al. [51,52]). The success of the surface sterilization method was confirmed by the absence of any microbial growth on PDA (potato-dextrose-agar) (Phyto Technology Laboratories, Lenexa, KS, USA) agar plates from the plating of the last root-washing water. Then, small fractions of sterilized root tissues (=0.5–1.0 cm^2^) were subsequently cultivated on PDA petri dish plates that contained a mixture of antibiotics (ampicillin and tetracycline 0.001% g mL^−1^ and kanamycin 0.005% g mL^−1^). FE were isolated from 90 root pieces (=30 root pieces from each pivotal root). This method of reducing the size and increasing the number of tissue fragments has been previously described as an appropriate method to estimate real values of FE diversity [53]. Plates were then incubated at room temperature for 2–3 weeks. After that time, emerging colonies were subcultured to obtain pure isolates. Pure isolates were grown on PDA plates (Phyto Technology Laboratories, Lenexa, KS, USA) at room temperature for one month for further DNA extraction.

### 4.3. DNA Extraction and Molecular Characterization of FE

All isolates used for DNA extraction were obtained from a monospore colony. Genomic DNA was extracted from the mycelial layer using a modification of the method described by Nicholson, et al. [54]. Fresh mycelium was ground on Mini-BeadBeater-16 (BioSpec, Bartlesbille, OK, USA). Then, the ground micelyum was suspended in an extraction buffer (10 mM Tris buffer pH 8.0, 10 mM EDTA, 0.5% SDS, NaCl 250 mM). A solution of phenol:chloroform:isoamyl alcohol (25:24:1) was added and mixed slowly for 5 min. The phases were then separated by centrifugation at 13.000 rpm for 10 min at 4 °C. DNA was precipitated from the aqueous phase with 2.0 volumes of isopropanol. The DNA was recovered by centrifugation at 13.000 rpm for 15 min at 4 °C. The pellet was then washed with 70% ethanol and resuspended in molecular biology grade water (Mo Bio Laboratories, Inc., CA, USA). ITS1-F KYO1 (CTHGGTCATTTAGAGGAASTAA) and ITS4 (TCCTCCGCTTATTGATATGC) primers were used for the identification of FE species. Partial amplification of the 18S rDNA gene (about 680 kpb) was performed with PCR reaction mixtures, each with 10–30 ng fungus genomic DNA, 10 pM of each primer, 1× SapphireAmp Fast PCR Master Mix (Takara, Otsu, Japan) and sterile water. PCRs were performed on a Techne TC-5000 thermocycler (Fisher Scientific, Burlington, NJ, USA) with the following program: 94 °C for 3 min, followed by 35 cycles of denaturation at 94 °C for 1 min, annealing at 54 °C for 30 s and primer extension at 72 °C for 1 min, completed with a final extension at 72 °C for 7 min. The amplified fragments were analyzed by 1% agarose gel electrophoresis. The PCR products were then sent to Macrogen (South Korea) for purification and sequencing. The sequences were assembled using the SeqTrace software. The assembled sequences (forward and reverse) were aligned with the program Codoncode Aligner. Sequence clusters with similarity of 97% or above were defined as operational taxonomic units (OTUs) using the CD-HIT program [55]. Consensus sequences were used for BLAST search at the NCBI (http://www.ncbi.nlm.nih.gov; accessed on 20 April 2022). FE frequency was expressed as the total number of fungal isolates corresponding to an Operational Taxonomic Unit (OTU). Diversity indexes such as Shannon and Simpson were also calculated for each location. Complementarily, the Chao and Bootstrap indexes were used as a richness estimator through the function specpool in the ‘vegan’ package. The relative abundance (in percentage) of the most dominant FE genera in the communities was also determined.

### 4.4. Edaphic Factors

Soil samples were collected from each plant (*n* = 10) in each location. All soil samples were collected in polyethylene bags (using a hand shovel) from a depth interval of 15–20 cm of the exposed soil face. Samples were transported to the lab and stored at 4 °C for further analysis. The following parameters were measured from each soil sample: soil water potential, soil moisture, pH and nutrients, including nitrogen (N), potassium (K) and phosphorous (P). Soil water potential (MPa) was measured with a Dew Point Potentiometer (Model WP4-T, Decagon Devices, Pullman, WA, USA). Soil moisture content (%) was determined by the ratio between water mass and dry mass of the soil, measured after drying the soil samples at 100 °C in the oven (Model FED 53-720, Binder, Tuttlingen, Germany). Analyses of pH and nutrients were carried out at Activation Laboratories Ltd., (Coquimbo, Chile), by using the methods described by Sadzawka, et al. [56].

### 4.5. Field Experiment

In order to experimentally assess whether FE frequency (total fungal isolates) is affected by different water regimes, 20 healthy plants of *A. chilensis* were selected in the more arid location along the gradient (=Huasco). FE frequency was used here as a proxy of abundance [57]. Plants were randomly assigned to one of two watering regimes: (1) no watering (control, *n* = 10 plants) and (2) 85 mm^3^ of watering (*n* = 10 plants). 85 mm^3^ of watering imitates the average rainfall in the Southern population of Quilimarí for the year of sample collection (year 2015). Quilimarí is the contrasting location (in terms of rainfall) to the location of Huasco (Figure 1). Plants were irrigated on two occasions, with an interval of 20 days between each event. Thirty days after the last irrigation, root material of watered and non-watered plants was collected in the field, and immediately transported into the lab. Once in the lab, the roots were subjected to sterilization and cultivation, as previously described, for further determination of FE frequency.

### 4.6. Statistical Analyses

Differences in relative abundance (%) of the most prevalent FE genera, fungal frequency (number of fungal isolates), Chao index (richness estimator), Bootstrap index (richness estimator), as well as Shannon and Simpson diversity indexes among locations were assessed with the non-parametric Kruskal–Wallis test, because data did not meet assumptions for parametrical tests. Differences in FE community composition among the three locations, and pair wise comparisons, were performed with a permutational multivariate analysis of variance (PERMANOVA) (9999 permutations) based on Euclidean distances, using the *adonis* function in the ‘vegan’ package. To visualize differences in FE communities among the locations, and aid in the interpretation of PERMANOVA results, we performed a non-metric multidimensional scaling (NMDS), based on the Euclidean matrix of dissimilarity, using the function *metaMDS* in the ‘vegan’ package. A distance-based redundancy analysis (dbRDA) was used to explore correlations between edaphic factors and FE communities. The *dbrda* function was performed with the selection criterion *ordistep* (‘vegan’ package) in order to obtain the most significant edaphic factors [58,59,60]. Differences in edaphic factors among the locations were determined with One-way ANOVAs. For the field experiment, differences in FE frequency between both watered and non-watered plants were assessed with a Kruskal–Wallis test. All statistical analyses were performed in R ver. 3.1.0 [61].

## Figures and Tables

**Table 1:** Best BLAST matches for OTUs of root endophytic fungi of *Aristolochia chilensis* plants from the three locations (H: Huasco; T: Totoralillo; Q: Quilimarí).

Location	Description	Identity (%)	No. of Isolates	Accession Number
Huasco	*Aspergillus ustus*	99.8%	2	HQ607918.1
	*Aspergillus versicolor*	100%	1	MT560203.1
	*Clonostachys* sp.	99.8%	3	MH864436.1
	*Fusarium oxysporum*	100%	41	MT560381.1
	*Fusarium* sp.	100%	19	MT447523.1
	*Hypocrea lixii*	99.5%	1	EF191308.1
	*Penicillium gladioli*	99.5%	3	DQ339568.1
	*Penicillium murcianum*	99.5%	3	KP016843.1
	*Penicillium polonicum*	100%	6	MT529240.1
Totoralillo	*Cladosporium ramotenellum*	100%	1	MT441591.1
	*Clonostachys rosea*	100%	14	MT441592.1
	*Diaporthe columnaris*	100%	2	MT441604.1
	*Epicoccum nigrum*	100%	1	MT441593.1
	*Fungal* sp.	100%	11	MT441613.1
	Fusarium decemcellulare	100%	3	MT441594.1
	*Fusarium oxysporum* 1	100%	3	MT441590.1
	*Fusarium oxysporum* 2	100%	39	MT530243.1
	*Fusarium* sp.	100%	115	MT441596.1
	*Fusarium sambucinum*	100%	8	MT441588.1
	*Hypocrea viridescens*	100%	2	MT441598.1
	*Meyerozyma caribbica*	100%	1	MT441599.1
	*Meyerozyma guilliermondii*	100%	14	MN592978.1
	*Penicillium crustosum*	100%	1	MT441603.1
	*Penicillium glabrum*	100%	5	MT441616.1
	*Penicillium murcianum*	100%	2	MT441602.1
	*Penicillium* sp.	100%	1	MT441612.1
	*Phialemoniopsis cornearis*	100%	9	MT441611.1
	*Phomopsis* sp.	100%	17	MT441605.1
	*Preussia australis*	100%	1	MT441614.1
	*Sarocladium spinificis*	100%	1	MT441606.1
	*Talaromyces amestolkiae*	100%	9	MT441607.1
	*Talaromyces minioluteus*	100%	1	MT441589.1
	*Talaromyces minioluteus*	100%	15	MT441589.1
	*Trichoderma atroviride*	100%	1	MT441609.1
	*Trichoderma breve*	97%	1	MT441608.1
	*Trichoderma lixii*	100%	1	MT441597.1
	*Trichoderma* sp.	100%	1	MT441610.1
	*Trichoderma virilente*	99.8%	8	NR_138447.1
Quilimarí	*Aspergillus flavipes*	99.8	1	EU645664.1
	*Fusarium oxysporum* 1	100	73	MT560381.1
	*Fusarium oxysporum* 2	99.4	117	ON081646.1
	*Fusarium oxysporum* 3	99.6	1	MH454072.1
	*Fusarium* sp.	99.5	2	HQ130713.1
	*Meyerozyma guilliermondii*	95.7	1	MH545918.1
	*Meyerozyma guilliermondii*	97.8	2	LC317638.1
	*Trichoderma* sp.	99.8	2	KX459438.1
	Uncultured *Clonostachys*	96.5	2	MK407059.1
	Uncultured fungus clone	99.7	2	KF800414.1

**Table 2:** Infection frequency and indexes of diversity of FE for the three locations where *Aristolochia chilensis* was collected (H: Huasco; T: Totoralillo; Q: Quilimarí). Values indicate means ± SE. Different letters indicate significant differences among locations (non-parametric Kruskal–Wallis test with Pairwise Wilcoxon Rank Sum Tests).

Indexes	H	T	Q
Chao	32.62 ± 4.62 a	54.43 ± 5.54 b	76.38 ± 1.63 c
Bootstrap	20.65 ± 3.02 a	54.26 ± 10.45 b	59.92 ± 1.16 c
FE frequency	7.90 ± 1.79 a	28.81 ± 6.03 b	20.20 ± 4.64 b
Shannon	0.88 ± 0.16 a	1.47 ± 0.19 b	0.91 ± 0.14 ab
Simpson	0.47 ± 0.09 a	0.58 ± 0.07 a	0.45 ± 0.06 a

**Table 3:** Permutational multivariate analysis of variance (PERMANOVA) of fungal endophyte communities in the three locations where *Aristolochia chilensis* was collected: Huasco (H), Totoralillo (T) and Quilimarí (Q) (*n* = 10 replicates per location). Pairwise PERMANOVAs between locations are also indicated.

	*df*	*SS*	*MS*	Pseudo-*F*	R^2^	*p*
Locations	2	934.7	467.3	2.63	0.16	0.008
Residuals	27	4790.1	177.4		0.83	
Total	29	5724.8			1	
H vs. T	1	100.5		0.74	0.03	0.740
H vs. Q	1	542.7		3.42	0.15	0.036
T vs. Q	1	758.9		3.17	0.15	0.004

**Table 4:** Distance-based redundancy analysis assessing edaphic factors that contribute to explain variations in fungal endophyte communities associated with roots of *Aristolochia chilensis* (asterisk represents a significant contribution). * *p* < 0.05, ns indicates non-significant differences.

Edaphic Factors	AIC	F	*p*
Soil water potential	82.04	1.63	0.03 *
Soil moisture	82.03	1.51	0.04 *
pH	81.42	1.27	0.15 ns
N	81.16	1.22	0.16 ns
P	81.18	0.99	0.46 ns
K	81.39	0.84	0.74 ns

## Data Availability

Data available on request. The raw reads of ITS sequences were deposited into the NCBI Sequence Read Archive database (BioProject accession No. PRJNA814878).

## Supplementary Materials

The following supporting information can be downloaded at: https://www.mdpi.com/article/10.3390/plants11111511/s1, Figure S1. Rarefaction curve for fungal endophytes isolated from roots of *Aristolochia chilensis* collected in three locations: Huasco, Totoralillo and Quilimarí; Table S1. Edaphic factors for three locations where *Aristolochia chilensis* was collected: Huasco (H), Totoralillo (T) and Quilimarí (Q).
