# Supplementary material for: Changes in Diversity and Community Composition of Root Endophytic Fungi Associated with Aristolochia chilensis along an Aridity Gradient in the Atacama Desert
Source: Plants (Basel). 2022 Jun 5;11(11):1511. doi: 10.3390/plants11111511 (PMC9182583; doi:10.3390/plants11111511)
Supplement: Supplementary file 1 [file plants-11-01511-s001.zip › plants-1664881-SI.pdf]

Supplementary materials

Figure S1. Rarefaction curve for fungal endophytes isolated from roots of *Aristolochia chilensis* collected in three locations: Huasco, Totoralillo and Quilimarí.

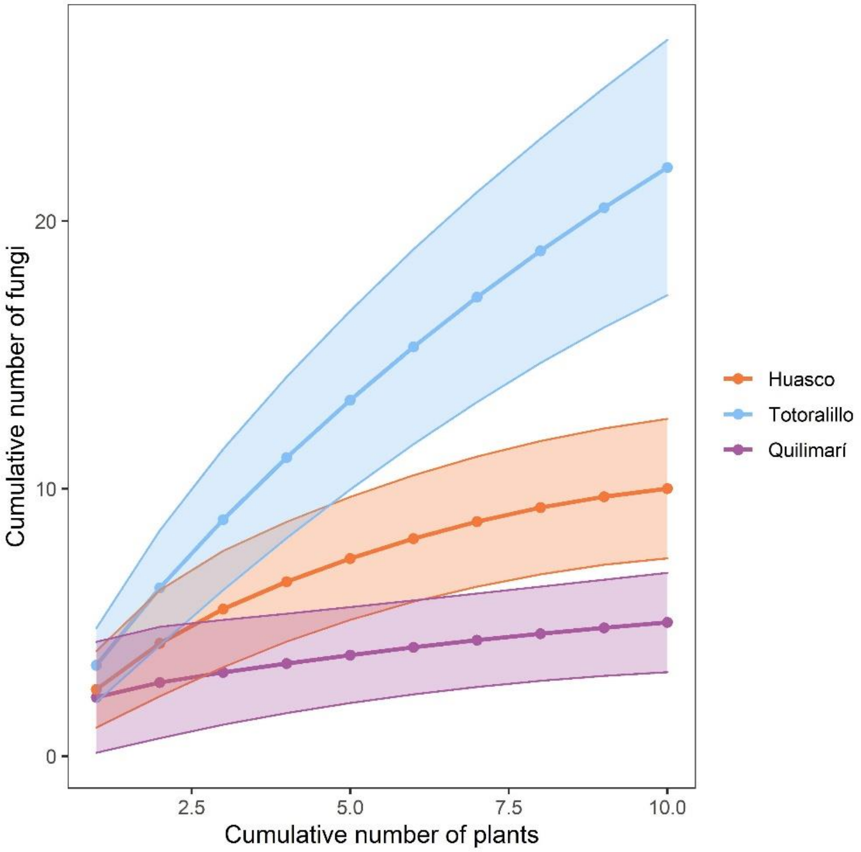

Table S1. Edaphic factors for three locations where *Aristolochia chilensis* was collected: Huasco (H), Totoralillo (T) and Quilimarí (Q). Values indicate the mean ± SE. Statistical results correspond to a One-way ANOVA with post-hoc Tukey. Different letters indicate significant differences among locations. For soil water potential, values near to zero indicate higher water availability.

**Edaphic factors**	**H**	**T**	**Q**	**d.f.**	***F***	***p***
Soil water potential	-27.8 ± 10.2 **a**	-4.26 ± 4.4 **b**	-35.7 ± 20.2 **a**	2.27	11.1	<0.001
Soil moisture	1.51 ± 0.9 **a**	3.53 ± 0.9 **b**	2.44 ± 0.8 **a**	2.27	12.1	<0.001
pH	7.97 ± 0.2 **a**	7.57 ± 0.5 **ab**	7.12 ± 0.7 **b**	2.27	5.77	<0.01
N	54.7 ± 11.1 **a**	43.1 ± 15.8 **a**	49.7 ± 12.7 **a**	2.27	1.88	0.170
P	58.5 ± 21.9 **a**	14.7 ± 9.6 **b**	36.5 ± 14.5 **c**	2.27	18.3	<0.001
K	328.5 ± 89.2 **a**	204.4 ± 39.1 **b**	264.6 ± 104.2 **ab**	2.27	5.67	<0.01
